# Lumping versus splitting: the need for biological data mining in precision medicine

**DOI:** 10.1186/s13040-015-0049-1

**Published:** 2015-06-11

**Authors:** Scott M. Williams, Jason H. Moore

**Affiliations:** 1 Department of Genetics, Institute for Quantitative Biomedical Sciences, The Geisel School of Medicine, Dartmouth College, One Medical Center Dr., Lebanon, NH 03756 USA; 2 Department of Biostatistics and Epidemiology, Institute for Biomedical Informatics, Perelman School of Medicine, University of Pennsylvania, Philadelphia, PA 19104-6021 USA

## Editorial

Biological data mining is playing an increasingly important role throughout the spectrum of biological and biomedical research with broad implications for the understanding of life science questions such as the tree of life and practical applications of such knowledge to improving human health. Perhaps nowhere is data mining needed more than the emerging discipline of precision medicine. The ability to predict individual risk of presenting with a disease or response to treatment is at the core of the concept of precision medicine, which is gaining ever-increasing levels of traction in the era of technology-driven measurement of biological systems. This has become especially important with the new Presidential initiative on precision medicine in the United States [1]. It is obvious to the readers of BioData Mining that this will require careful analyses of large and often complex data sets to best translate information into increasingly individualized risk. Here we ask why improved and appropriate data mining is not only positive but a vast improvement on most current analyses of genomic data. The answer lies to some extent in elucidating the present practice of -omic analyses and how we will need to expand it.

Many current -omic approaches rely on univariate and linear analyses that can often miss the underlying architecture of complex traits. For example, univariate analyses of single genetic markers for association with disease risk, prognosis, or drug response that are the analytical standards for genetic analyses of human disease, and have been promoted as a means to develop personalized or more recently precision medicine, make many assumptions about architecture. Given the interest in precision medicine, it is important to ask explicitly what is being assayed in these types of studies that have been argued, incorrectly we believe, as the precursors to precision medicine.

Most human geneticists study the association of genetic variants, be they common or rare, assessed across moderate to large samples of cases and controls. The effect of each allelic substitution is then measured as it associates with a particular phenotype. These estimates can provide useful population level risks; however, they are simply the average effect of an allelic substitution across the population, not necessarily predictive of results in an individual or a subgroup. The concept of average allelic effect is one that is well developed in quantitative genetics, but by its very name is suggestive not of precision medicine but of average medicine. Hence, it is possible in a large outbreeding population with multiple types and levels of environmental exposure that an average odds ratio can have large variances, which are often reported as 95 % confidence intervals. However, some people with a “risk allele, as defined by the population average, will not have an increased risk. At the extreme it is even possible to have opposite effects. For example, it is well recognized that increasing levels of salt intake can on average result in higher blood pressure and subsequent sequelae, including end stage organ disease. But as much as 10 % of some populations may have inverse salt sensitivity or an increase in blood pressure with decreasing salt intake. This illustrates that lowering salt is not a universal good [2]. On top of this there is some evidence that different genes associate with inverse salt sensitivity than those that associate with canonical salt sensitivity, making direct comparisons impossible [3]. Of course, this limitation is not unique to genetic analyses as environmental exposures are also usually dealt with primarily as univariate. How we navigate such issues is important in achieving more precise medicine.

An example from a non-disease outcome may further illustrate this. Assume we want to plan a manufacturing strategy for men’s pants. Would we simply identify the average pant size of all men in the US? If we only manufacture pants of the average waist size, say 34 in., under the premise that we have carefully calculated the average pant size in a very large cohort, the pants would only find a market in those close to the mean. This would make a particularly bad strategy and would be a terrible business model that no one would seriously consider. Such an approach is neither individualized nor rational. Yet, this may be akin to a one drug fits all people and even perhaps all people of a single genotype at a single site. Although there are cases in which this may work, it cannot work universally. An alternative strategy might be to stratify men by age, weight, height and physical activity as averages within each of these subgroups will be much more likely to provide better estimates of waist size as the variance within each strata are surely going to be smaller than in the population as a whole, providing increased precision. The unstated idea in this approach is to redefine subgroups such that the variance of the group is minimized as much as practical.

Alternatively, splitting into multiple groups may be less productive than we argue above. That is, in examining disease presentations and etiologies, it was argued decades ago that disease phenotypes may to a large extent reflect limitations of the clinician who sees a patient [4]. In such cases, syndromes with many presenting phenotypes may be dealt with by a specialist based on his or her limited perspective. In the modern era we hope that carefully curated electronic medical record data can be used to more effectively define syndromic cases and mitigate this problem, but it needs to be carefully considered that in splitting we do not split apart diseases that share a substantial portion of their etiology but are misclassified due to clinical bias. Interestingly, in this paper by McKusick it is described how causative genetic loci may be a way to partially address this in that genetic information will be a means to correct for excessive splitting, but this was argued on the bases of Mendelian diseases. In the case of more complex diseases it may be problematic to use genetic heterogeneity as even with strongly associating loci, context may be the most critical factor. Such careful analyses is a necessary prerequisite to precision medicine.

We therefore are arguing that by appropriate mining and subdivision of disease presentation by context, be it environmental, genetic or epigenetic, we can define subgroups with smaller variances, so that prediction of disease or treatment response can have utility. This approach recognizes that even though individualization per se may be impossible, by using large enough data and appropriate analyses, we may be able to define small enough groups to increase precision significantly in the practice of medicine. Biological data mining thus has a very important role to play in the President’s precision medicine initiative and across the many smaller basic science and clinical studies to understand the delivery of healthcare to individuals.
